# An Innovative Management in the Diagnosis of Mediastinal Masses

**DOI:** 10.1111/1759-7714.70029

**Published:** 2025-03-19

**Authors:** G. Messina, D. G. Pica, G. Vicario, N. M. Giorgiano, R. Mirra, V. Di Filippo, F. Capasso, F. Panini D’Alba, R. Vinciguerra, B. Leonardi, M. A. Puca, M. Grande, M. Marvulli, M. Messinó, M. Ciaravola, L. Ferrante, G. Vicidomini, A. Fiorelli

**Affiliations:** ^1^ Thoracic Surgery Unit, Università degli Studi della Campania “Luigi Vanvitelli” Napoli Campania Italy; ^2^ Anesthesia and Intensive Care Unit, Università degli Studi della Campania “Luigi Vanvitelli” Napoli Campania Italy

**Keywords:** biopsy, chest tumors, intraoperative ultrasound (IUS), malignant mediastinal mass, mediastinal lesions, mediastinoscopy, mediastinum, video‐assisted thoracoscopic surgery (VATS)

## Abstract

**Introduction:**

The mediastinum is a complex anatomical region that contains vital structures such as the great vessels, heart, esophagus, and trachea. Mediastinal masses include a wide range of lesions, both malignant and benign. Our study aimed to evaluate whether the combination of intraoperative ultrasound (IUS) and VATS can allow obtaining an adequate, correct, and safer diagnosis in patients with a mediastinal mass, especially in severely ill patients affected by heart failure, renal failure, advanced oncological stage, and respiratory failure.

**Material and Method:**

This single‐center, retrospective, observational study included 298 consecutive patients with mediastinal mass, evaluated between March 2018 and December 2024 at the Thoracic Surgery Department of Vanvitelli University of Naples. All patients underwent biopsies of mediastinal masses via VATS with IUS. Mediastinal masses were classified based on their ultrasound characteristics, allowing precise identification of the site and solid part of the lesions for biopsy. No significant complications were reported.

**Results:**

A total of 298 patients underwent VATS for mediastinal mass biopsy. About 185 of these patients underwent biopsies via VATS with IUS. All were under general anesthesia with single‐lung ventilation. A specific diagnosis was obtained in all patients who underwent biopsies via VATS with IUS (185/185), with lymphoma being the most common entity (58.6%), followed by germ cell tumors (24.3%) and thymic carcinoma (12.4%). A significant difference in diagnostic accuracy, specificity, and sensibility was found between the group that used IUS versus the group in which no IUS was used (100%vs. 93%, 99.8% vs. 94%, 98.5% vs. 90.5% respect).

**Conclusion:**

IUS‐guided biopsy allows for correct, safe, and precise identification of mediastinal lesions, establishing IUS as the “Gold Standard” for procedure guidance when the target lesion is adequately visualized.

## Graphical abstract

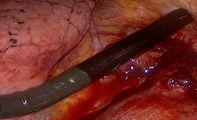

## 1 Introduction

The mediastinum is a complex anatomical region that contains vital structures, including the great vessels, heart, esophagus, and trachea. Mediastinal masses constitute a broad spectrum of lesions that can be malignant or benign. Anterior mediastinal masses (AMM) are heterogeneous in etiology and origin, including epithelial, mesenchymal, hematopoietic, lymphoid, and metastatic neoplasms [1, 2, 3, 4]. Some typically benign lesions do not require treatment, while patients with clinical symptoms or suspected malignant AMM should be treated promptly [5, 6, 7]. Treatment strategies include surgery, chemotherapy, and/or radiotherapy based on the specific pathological diagnosis and clinical staging [8, 9]. Therefore, a precise and accurate histopathological pre‐treatment diagnosis is essential for patients. There are several strategies for taking samples from mediastinal lesions for histopathological diagnosis, such asmediastinotomy, mediastinoscopy, bronchoscopy, open surgical biopsy, video‐assisted thoracoscopic surgery (VATS) and CT‐guided or ultrasound‐guided transthoracic biopsy [10, 11, 12]. Each technique has advantages and disadvantages in terms of accuracy, invasiveness, cost, and risk [13, 14]. Although transparietal needle aspiration [11] is a valid tool for the diagnosis of AMM, thanks to the minimally invasive approach, it is not considered the first choice by recent guidelines; therefore, a surgical biopsy is often preferred to obtain an adequate amount of tissue for diagnosis [15, 16, 17]. The role of VATS is fundamental [18] because it combines low invasiveness with the possibility of obtaining a histological sample [19, 20]. Our study aimed to evaluate whether the combination of intraoperative ultrasound (IUS) and VATS can allow obtaining an adequate, correct, and safer diagnosis in patients with a mediastinal mass, especially in severely ill patients affected by heart failure, renal failure, advanced oncological stage, and respiratory failure.

## 2 Materials and Methods

This single‐center, retrospective observational study includes all consecutive patients evaluated between March 2018 and December 2024; all consecutive patients admitted to the Department of Thoracic Surgery of the Vanvitelli University of Naples underwent biopsies of mediastinal masses in VATS and with IUS. Patients underwent general anesthesia and endotracheal intubation with a double‐lumen tube and contralateral single‐lung ventilation; the correct position of the orotracheal tube was confirmed by fiberoptic bronchoscopy with unilateral pulmonary ventilation.

**FIGURE 1:**
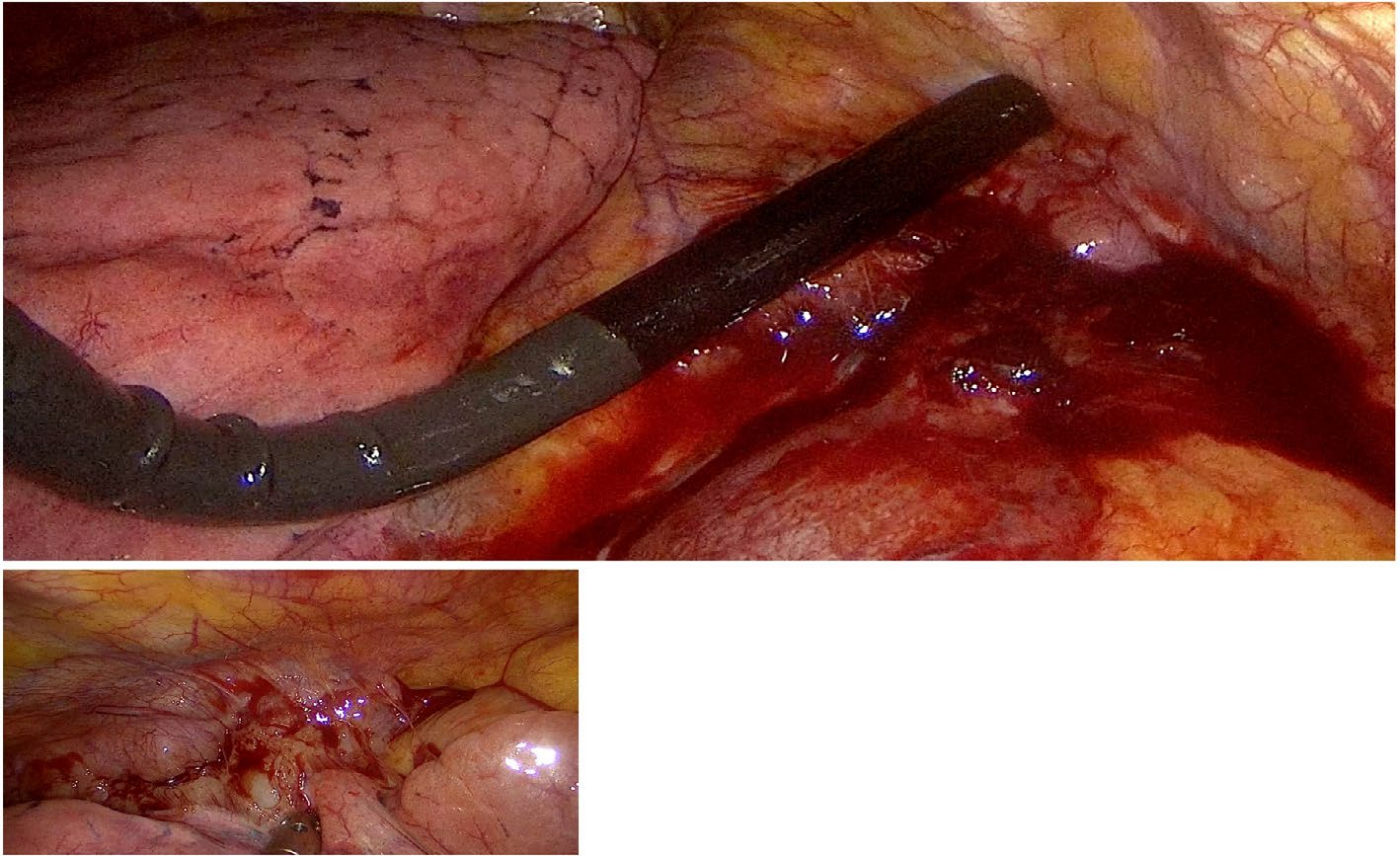
The probe was inserted into the chest through the uniportal incision, and the AMMs were explored using ultrasound.

**FIGURE 2:**
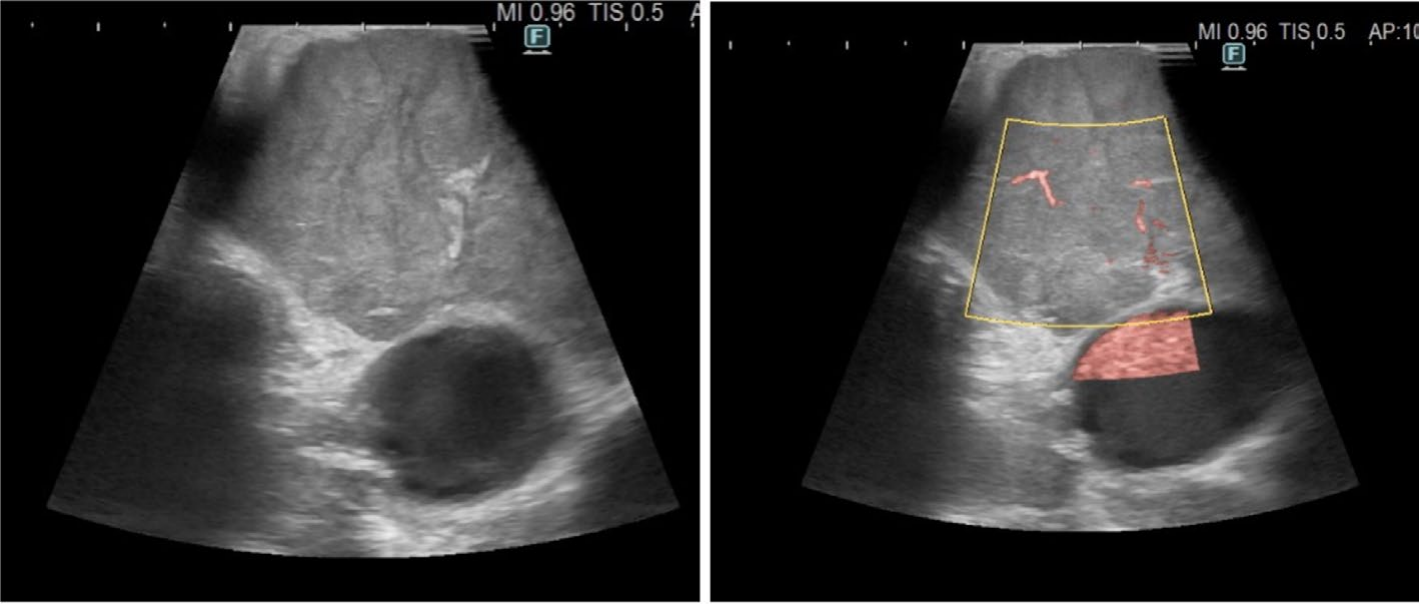
AMMs were classified based on their ultrasound characteristics, including shape, echogenicity, short‐axis diameter, margin, increased color Doppler flow, presence or absence of coagulative necrosis, presence or absence of calcification, nodal conglomeration, and vascularization of the lesion.

**FIGURE 3:**
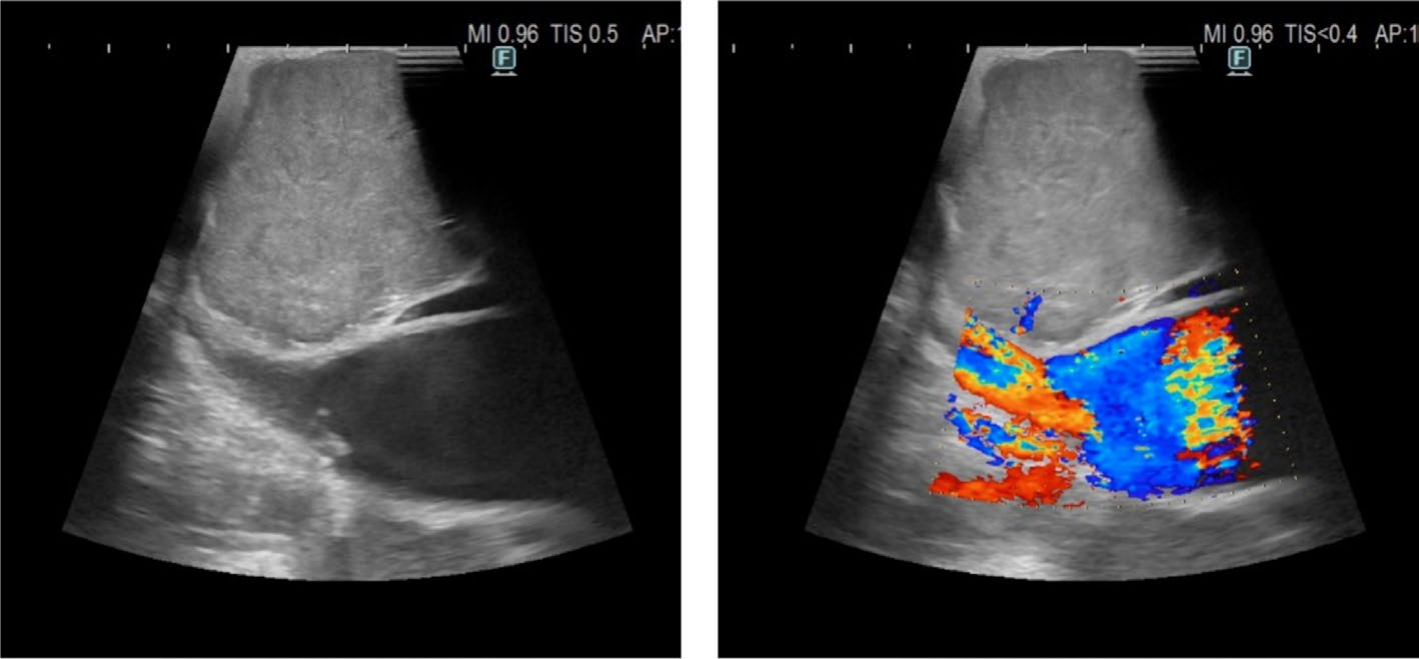
The ultrasound characteristics of mediastinal masses were: Large hypoechoic lesions, inhomogeneous echostructure, increased vascularization in the intra‐ and perinodular color Doppler, presence of hyperechoic spots, and contextual anechoic liquefactive areas.

**FIGURE 4:**
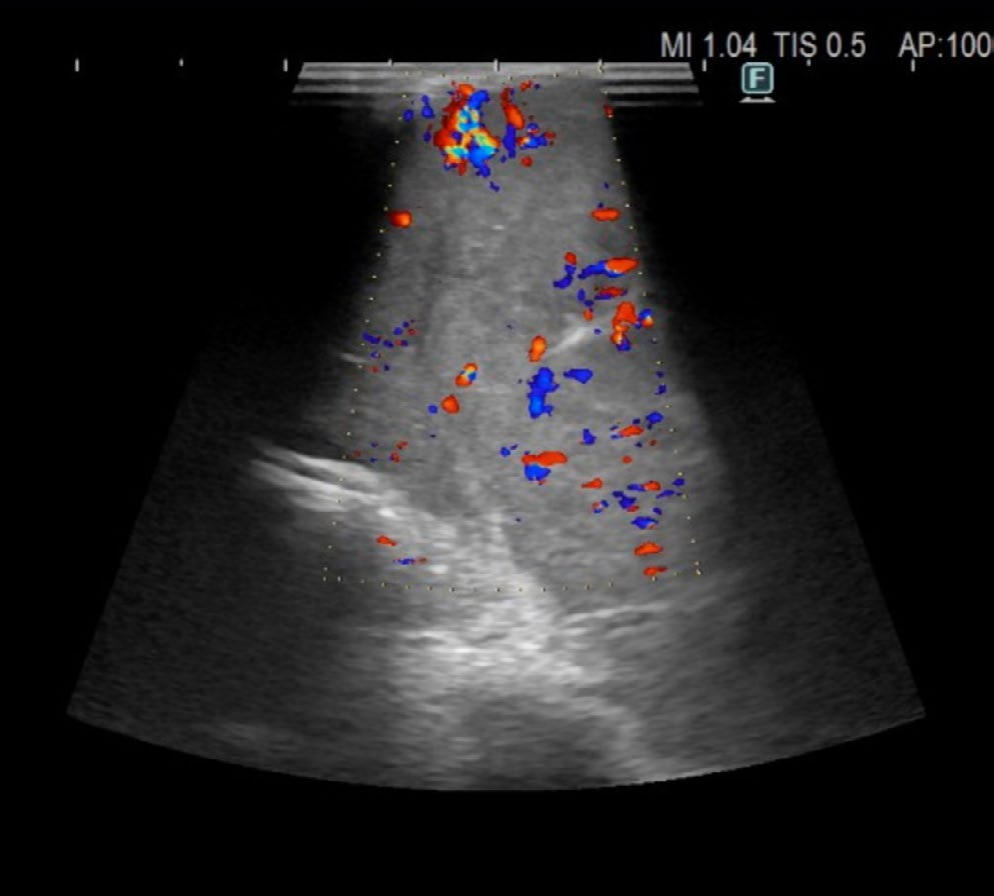
The ultrasound characteristics of mediastinal masses show: Increased vascularization in the intra‐ and perinodular color Doppler.

After being anesthetized, the patient was positioned in the lateral decubitus position. The uniportal incision was made at the fifth intercostal space, anterior to the latissimus dorsi and posterior to the pectoralis major. The size of the uniportal access incision ranged from 2 to 4 cm (mean 3 cm). Chest exploration revealed the presence or absence of pleural adhesions and pleural nodules. The probe was inserted into the chest through the uniportal incision, and the AMMs were explored using ultrasound. The AMMs were subsequently labeled, and their characteristics were classified under ultrasound guidance. The ultrasound processor used for the examination of the AMM was the BK 5000, and through uniportal access, a sterile intracavitary laparoscopic probe was introduced, 38 cm long and 10 mm in diameter, with a flexible tip, equipped with a convex matrix transducer with frequencies between 4 and 12 MHz (Figure 1). The lung under examination was collapsed to facilitate the movements of the instruments and the ultrasound probe inside the pleural cavity; by applying pressure on the visceral pleura using the probe, a desufflation of the lung was obtained to eliminate the air around the affected lung during the exam. The probe was positioned perpendicular to the mediastinal tissue containing the mass. A warm sterile saline solution allows for the best ultrasound transmission. No changes in standardized surgical procedures were determined by the use of the probe. IUS prolonged the operative time by approximately 11 min (range 8–12 with 95% confidence interval [CI] [9.57–10.19]) compared to surgery without IUS. The shape of the tumor was assessed with a small ultrasound probe introduced through the trocar, equipped with a function that allows a 180° rotation from right to left and from top to bottom, thanks to a handle that acts as a grip and controls its movement, allowing visualization of the internal mediastinal mass. AMMs were classified based on their ultrasound characteristics related to shape, echogenicity (heterogeneous or homogeneous), short axis diameter, margin (distinct or indistinct), increased color Doppler flow, absence or presence of the sign of coagulative necrosis, absence or presence of calcification, nodal conglomeration, and vascularization of the lesion (Figure 2). The ultrasound characteristics of mediastinal masses were large hypoechoic lesions, inhomogeneous echostructure, increased vascularization in the intra‐ and perinodular color box, presence of hyperechoic spots, and contextual anechoic colliquated areas [21, 22] (Figures 3 and 4).

**FIGURE 5:**
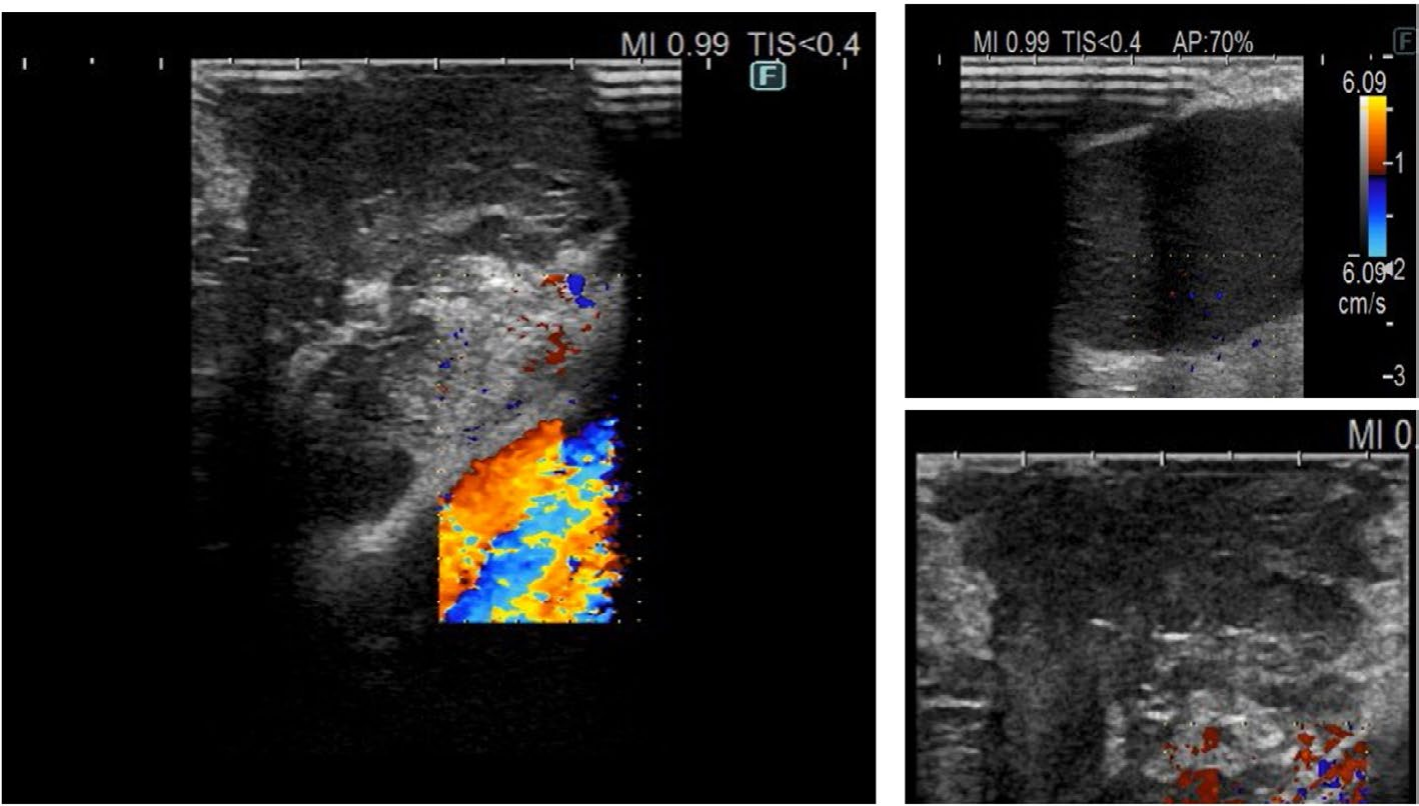
Using intraoperative ultrasound, we identified the exact site and solid part of the lesion where to perform the biopsies and indicated the depth and direction for the forceps.

Using IUS, we identified the exact site and the solid part of the lesion where to perform the biopsies, indicating the depth and direction of the forceps (Figure 5). Furthermore, with the use of IUS, we identified in real time the main intrathoracic blood vessels and the internal thoracic arteries, as well as any major collateral veins that could have formed in the event of obstruction of the superior vena cava and therefore, we avoided injuring blood vessels during the biopsy, also avoiding sampling necrotic material that is inadequate for diagnosis [23, 24, 25].

In the group of patients undergoing IUS, we selected the optimal site to perform a biopsy; after opening the mediastinal pleura by forming a small incision, the neoplasm was then sampled with biopsy forceps and sent to pathological anatomy, ensuring an adequate quantity of tissue also for immunohistochemistry. Finally, careful hemostasis was performed before closing the chest and positioning the endopleural drainage tube. The patient was returned to the ward after a short period of observation in the operating room, and a chest x‐ray was performed to exclude a pneumothorax. Postoperative pain was also quantified using a visual analog scale (VAS) with levels ranging from 0 to 10, immediately after surgery. To achieve optimal postoperative pain control, an ultrasound‐guided Erector Spinae Plane (ESP) block was performed with a bolus of ropivacaine 1% and dexamethasone. Patients did not experience significant complications such as bleeding and/or pneumothorax [26, 27].

## 3 Statistics

**FIGURE 6:**
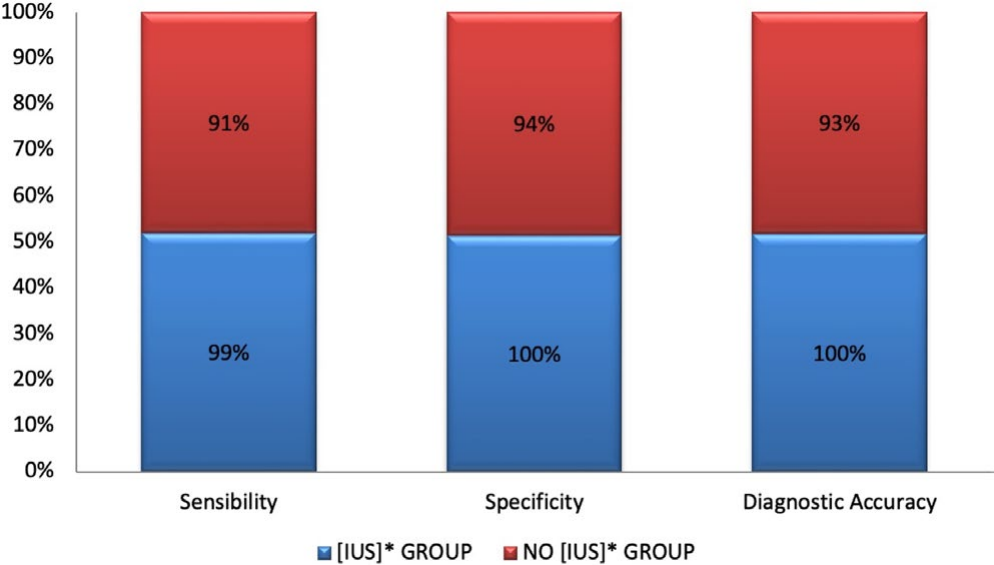
A significant difference in diagnostic accuracy was found between that used intraoperative ultrasound ([IUS] GROUP) versus the group in which no intraoperative ultrasound was used (NO [IUS] GROUP) 100% versus 92.5%. The visualization and identification of AMM significantly improved after the use of IUS (specificity) 99.8% versus 94%; (sensibility) 98.5% versus 90.5%.

Categorial data was expressed as percentages. Continuous data with normal distribution was expressed as mean ± standard deviation; otherwise, it was expressed as median. The difference in lesion size between a group with IUs and a group without IUS was analyzed via the Independent‐Samples *T* test. A significant difference in diagnostic accuracy, specificity, and sensitivity was found between the group that used IUS versus the group in which no IUS was used (100%vs. 93%, 99.8% vs. 94%, 98.5% vs. 90.5% respect) (Figure 6).

## 4 Results

This is a single‐center retrospective observational study including 298 consecutive patients with mediastinal mass, evaluated between March 2018 and December 2023 at the Thoracic Surgery Unit of the Vanvitelli University of Naples.

Inclusion criteria: patients with mediastinal lesions larger than 35 mm, detected by contrasted chest CT and showing increased uptake on FDG‐PET/CT (SUVmax [mean ± SD] 7.4 ± 4.2); patients without histological diagnosis after bronchoscopic biopsy and transbronchial needle aspiration (FNA); normal coagulation.

Exclusion criteria: uncorrectable coagulation abnormalities, INR > 1.4; platelets < 50 000/mL; aPTT > 1.5 times higher than the reference range.

Of the total number of selected patients, 113 were operated on in the operating block not equipped with an IUS probe and were therefore used as a control group (NO [IUS] GROUP). The remaining 185 patients all underwent VATS and IUS for mediastinal mass biopsy ([IUS] GROUP).

In the NO [IUS] GROUP, 68 patients were male (60.1%) and 45 were female (39.9%). In this group, 8 patients (7%) were observed with an incidental finding of AMM without symptoms during a routine physical examination, while the others showed symptoms such as 48 patients with cough (42.5%), 52 patients with chest pain (46%), 8 patients with fever (7%), and 3 patients with dyspnea (2.7%) (Table 1).

**TABLE 1:** Characteristics of the NO [IUS] GROUP.

Variables	Number of subjects (*n* = 113)
Gender	
Male	68 (60.1%)
Female	45 (39.9%)
Signs and symptoms	
Chest pain	52 (46%)
Cough	48 (42.5%)
Fever	8 (7%)
Dyspnea	3 (2.7%)
Dysphagia	2 (1.8%)
Haemopthysis, pneumothorax	0

In the [IUS] GROUP, 110 patients were male (59.4%) and 75 were female (40.5%). In this group, 7 patients (4%) were observed with incidental discovery of AMM without symptoms during routine physical examination, while the others showed symptoms such as 83 patients with cough (46.6%), 86 patients with chest pain (48.6%), 28 patients with fever (15.6%), 16 patients with dyspnea (9.3%), 7 patients with swelling of the head and face (4%), 4 patients with hoarseness of the voice (2.5%), 5 patients with dysphagia (2.8%) and 3 patients with haemoptysis (1.5%) (Table 2).

**TABLE 2:** Characteristics of the [IUS] GROUP.

Variables	Number of subjects (*n* = 185)
Gender	
Male	110 (59.4%)
Female	75 (40.5%)
Signs and symptoms	
Chest pain	86 (48.6%)
Cough	83 (46.6%)
Fever	28 (15.6%)
Dyspnea	16 (9.3%)
Swelling of the head end of the face	7 (4%)
Dysphagia	5 (2.8%)
Hoarseness of the voice	4 (2.5%)
Haemopthysis	3 (1.5%)

Ultrasound visualization of mediastinal lesions located in the anterosuperior compartment was successful in all patients (100%) (Figure 5).

Preoperative evaluation included: cardiac examination, electrocardiogram (ECG) and echocardiography; functional respiratory tests, standard spirometry, and arterial blood gas analysis.

IUS was performed in all patients. Procedures were performed under direct IUS guidance. The IUS allowed real‐time visualization of the exact and appropriate site where to perform the biopsy; Color Doppler allowed the large vessels, collaterals, and tumor vessels to be clearly and easily highlighted, avoiding their laceration during the biopsy. The size of the lesion was large in both the short axis (median [IQR] mm = 61 [46.3–108.8]) and long axis (median [IQR] mm = 108.5 [92–135.3]).

Based on the final diagnosis, lymphoma 108 (58.6%) was the most common entity, followed by thymic carcinoma 43 (23.5%) and germ cell tumor 25 (13.5%).

The percentage (185/185) 100% of patients who underwent biopsies via VATS with IUS obtained a specific diagnosis with sufficient information for therapy.

The ultrasound characteristics of the lesions: hypoechoic ovoid shape, regular margins (78.7%), irregular margins (69%), inhomogeneous structure (71%), colliquated areas (43%), floating echoes (55%), thin walls (43%), thickened walls (26%), internal calcifications (73%), increased intralesional color box signal (33%) and perilesional (41%) (Table 3).

**TABLE 3:** The ultrasound characteristics of anterior mediastinal masses (AMM).

Tumor size	
Anterior mediastinal masses (ANM)	Short axis (median [IQR] mm = 61 [46.3–108.8])
	Long axis (median [IQR] mm = 108.5 [92–135.3])
Ecostructure	
Regular margins	145 (78.7%)
Inhomogeneous structure	131 (71%)
Irregular margins	128 (69%)
Floating echoes	102 (55%)
Thin walls	80 (43%)
Perilesional color box signal	76 (41%)
Intralesional color box signal	61 (33%)
Thickened walls	48 (26%)
Histological characteristics	
Internal calcifications	135 (73%)
Colliquated areas	80 (43%)
Primary oncological disease	
Lymphoma	108 (58.6%)
Thymic carcinoma	43 (23.5%)
Germ cell tumor	25 (13.5%)

The mean operative time was 25.4 ± 5.2 min.

Careful hemostasis was then performed before chest closure. All patients were returned to the ward after a short period of observation in the operating room of approximately 20 minutes, and a chest x‐ray was performed to exclude a pneumothorax. Then, patients were referred for appropriate chemotherapy and/or radiotherapy within 30 days of biopsy.

The group of patients in whom ultrasound was not used (NO [IUS] GROUP) includes 113 patients, of which 6 patients whose samples contained a large necrotic area could not obtain a diagnosis; therefore, they underwent a new biopsy (two squamous cell carcinoma of the thymus and four lymphomas) for a therapeutic program. The other 10 (20.8%) cases were accepted as diagnostic biopsy failures based on the final diagnosis. Patients showed no symptomatic complications such as bleeding, pneumothorax, or hemoptysis after biopsy. A significant difference in diagnostic accuracy, specificity, and sensibility was found between the group that used ultrasound versus the group in which no ultrasound was used (95%vs. 77%, 96% vs. 61%, 98% vs. 80% respect).

## 5 Discussion

AMMs include multiple diseases: primary and secondary, neoplastic and infectious, malignant and benign. Early diagnosis and treatment are crucial in the management of patients with malignant AMM. Saito et al. two decades performed ultrasound‐assisted biopsies of mediastinal masses for the first time [28, 29, 30]. Nowadays, B‐mode ultrasound is rarely used in the study of the mediastinum, although this method is a valid diagnostic tool complementary to other techniques such as chest x‐ray, chest CT, and chest MRI. However, ultrasound of the mediastinum is much more sensitive compared to standard chest radiography in the diagnosis of mediastinal tumors [31]. Considering chest CT as a reference method, ultrasound, compared to radiography, shows greater sensitivity for each site: paratracheal region 89% versus 69%, aorta‐pulmonary window 81% versus 62%, prevascular region 92% versus 46%, subcarinal region 69% versus 31%, versus 67%, supra‐aortic region 98% versus 67%, and pericardial region 100% versus 67% [32, 33]. Obviously, ultrasound of the mediastinum is less effective than CT in detecting pericardial, supra‐aortic, and perivascular lesions (sensitivity 98%–100%) and some sites, such as the posterior mediastinal and paravertebral regions, can only be evaluated by MRI and CT [34, 35] The use of ultrasound, thanks to the possibility of performing it in different contexts, from the radiology department to the patient's bed and to the operating room. In the diagnosis of mediastinal lesions, it is very widespread [36, 37]. IUS can provide useful information in the complex evaluation of mediastinal masses located in the anterior (prevascular) and posterior compartments of the mediastinum. It also offers the possibility to guide biopsies in real time in such clinical scenarios, with several advantages over CT. IUS guaranteed rapid localization of the mass in the operating room, it does not use ionizing radiation to the benefit of the patient and color Doppler allowed the vessels and vascularized tumor tissue to be correctly identified to avoid intraoperative hemorrhages [38, 39]. An accurate histological diagnosis is essential for a correct therapeutic plan and also for preoperative neoadjuvant therapy, an inconclusive diagnosis delays specific therapy [40, 41]. The most common cause of diagnostic failure is tumor necrosis, sampling error, or inadequate sampling [42]. IUS shows non‐liquefied necrosis, effectively assessing tissue necrosis and viability and identifying perfused areas resulting in increased diagnostic accuracy. In the present study, the visualization and identification of areas of internal necrosis significantly improved by 100% after the use of IUS. However, IUS guidance allows effective real‐time monitoring of the exact site where the biopsy should be performed, providing precise indications regarding the exact angle and depth of the forces; Color Doppler also allows you to evaluate the vascularity of the lesion and the vascular structures, preventing possible bleeding, this method therefore allows critical and advanced cancer patients to be subjected to a mediastinal biopsy, thus avoiding complications and possible re‐operations [43]. Surgical procedures have an accuracy of up to 100% and in some cases could simultaneously establish the diagnosis and provide treatment. All surgical procedures in VATS with IUS; require general anesthesia and short hospitalization, no complications are observed in all patients [44, 45, 46]. The success of a mediastinal biopsy depends on many factors including the clinical circumstances, the size and location of the lesion, the presence of comorbidities, the operator's experience in IUS, and the institution's willingness to allow performing this diagnostic method [16, 47, 48]. However, this study has several limitations, being a retrospective observational single‐center study; the application of the IUS was not random and therefore was based on the specialist's recommendation and the patient's agreement.

## 6 Conclusion

In the era of minimally invasive approaches, VATS together with IUS allows the correct, safe, and precise identification of masses and vessels; this method can be performed quickly and with no perioperative complications, with a diagnostic yield of 100% thanks to the satisfactory quality of tissue obtained and the possibility of evaluating the malignancy of the pathology, thus allowing a timely and appropriate treatment of this oncohematological pathology. Therefore, IUS is considered the “Gold Standard” for procedure guidance if the target lesion can be adequately imaged; however, further studies are necessary to strengthen the results we obtained.

## 7 Author Contributions

All authors contributed to design of the study. G. Messina and D. G. Pica contributed to the conception of the study. G. Vicario, V. Di Filippo, and F. Capasso contributed to the drafting of the article. N. M. Giorgiano, F. Panini D'Alba, R. Vinciguerra and B. Leonardi contributed to data collection and imaging analysis. R. Mirra, M. A. Puca, M. Grande, M. Messinó and M. Marvulli participated in data analysis and interpretation and led the revision of the article. M. Ciaravola, L. Ferrante, G. Vicidomini and A. Fiorelli supervised the study. All authors reviewed and approved the final manuscript.

## Ethics Statement

The authors are accountable for all aspects of the work in ensuring that questions related to the accuracy or integrity of any part of the work are appropriately investigated and resolved. The study was led in compliance with the principles of the Declaration of Helsinki; written informed consent was obtained from all participants during preoperative communication, and the protocol was approved by the Ethics Committee of the University of ‘Luigi Vanvitelli’ of Naples (32655/2021).

## Conflicts of Interest

The authors declare no conflicts of interest.

## Data Availability

Authors can confirm that all relevant data are included in the article.
